# Smartphone-Based Augmented Reality Applications for Percutaneous Procedures: A Pragmatic Step Toward Clinical Use

**DOI:** 10.1007/s00270-025-04087-7

**Published:** 2025-06-17

**Authors:** Uli Fehrenbach

**Affiliations:** Department of Radiology, Charité Universitätsmedizin Berlin, Augustenburger Platz 1, 13353 Berlin, Germany

Augmented reality (AR) has found its way into everyday life in the entertainment sector, ranging from mobile gaming to social media filters. Besides its qualities in lifestyle media, AR technologies are now increasingly explored for its potential in medical applications. AR tools have long promised to enhance image-guided interventions, yet the final step from proof of concept to practical clinical use is lacking. Saccenti et al. present a compelling and at the same time pragmatic approach: a smartphone-based AR system with integrated needle guidance for percutaneous procedures [1]. Unlike other high-end AR solutions requiring head-mounted displays or external workstations, this setup is both technically minimalistic and operationally efficient. It uses a commercially available smartphone, a 3D-printed case, and an FDA-cleared disposable needle guide. Trajectory planning and real-time feedback would be performed directly on the device, eliminating the need for additional hardware and therefore also impeding extra costs.

The authors report a high accuracy in their phantom study when using their AR smartphone tool: Median deviation from the target was only a few millimeters without using any intraprocedural imaging in out-of-plane punctures. Interestingly, the benefit was consistent across operator experience levels, suggesting that the system may flatten the learning curve and could therefore also standardize outcomes what would be a desirable feature in interventional radiology training.

This work represents another possible shift from technology-driven to workflow-oriented AR development. As smartphone guiding exists already for quite a while, previous approaches, including those using smartglasses, often remained confined to research due to complexity, costs, or limited possibilities for integration into practice [2, 3]. Saccenti et al.’s approach focuses on simplicity and translation into clinical routine: intuitive single-user application, reduced operator dependence, and compatibility with existing infrastructure.

The implications of AR in interventional radiology are relevant beyond academic centers. Especially in resource-limited settings, where access to CT fluoroscopy or cone-beam CT is limited, smartphone-based AR guidance could enable safer and more accurate needle placement with minimal capital investment. Moreover, as shown before, successful implementation of augmented or mixed reality into clinical routine depends not only on spatial accuracy of the device but also on seamless data integration and workflow compatibility [4].

Nonetheless, several limitations of the study deserve careful consideration. The most important limitation lies in the experimental setup that reflects idealized conditions: immobile phantoms without (respiratory) motion, tissue deformation, or anatomical variability. These factors, however, are completely different to real-life interventions, particularly in organs such as the liver or lung, where breathing and organ mobility significantly impact targeting accuracy and also increase the procedure’s complexity. As such, the system’s suitability for complex, dynamic interventions, particularly in organs affected by respiration, appears limited. For static targets however, the method is promising and may improve accuracy while reducing radiation exposure at the same time. Another limitation for the translation into clinical routine is that the system still relies on prior CT segmentation, which may pose workflow challenges in acute or time-sensitive interventions. Inaccuracies of the pre-interventional segmentation would also lower the accuracy of this specific tool and must be carefully avoided. While this potentially time-consuming step was completed externally in this study, its clinical deployment would benefit from on-device or automated CT segmentation tools, another frontier where artificial intelligence is already becoming essential.

In summary, Saccenti et al. demonstrate that AR guidance can be both technically elegant and pragmatic enough to be potentially clinically feasible. Their work is another step in the translation of interventional AR into clinical routine: from experimental showcases to practical tools. The inevitable final step is now to critically evaluate the utility and robustness in the real-world clinical environment.
